# From Quantifying to Managing Food Loss in the Agri-Food Industry Supply Chain

**DOI:** 10.3390/foods10092163

**Published:** 2021-09-13

**Authors:** Eva M. Sánchez-Teba, Germán Gemar, Ismael Pablo Soler

**Affiliations:** 1 Department of Economics and Business Administration, University of Malaga, 29071 Malaga, Spain; ggemar@uma.es; 2 Department of Applied Economics (Statistics and Econometrics), University of Malaga, 29071 Malaga, Spain; ipsoler@uma.es

**Keywords:** SciMAT, food loss and waste, food supply chain, agri-food companies, sustainable development goals, bibliometric analysis

## Abstract

The significant contradiction of food waste and food insecurity that preoccupies society today is growing increasingly important. It is estimated that one-third of all food produced globally is either lost or wasted. In a world where almost one billion people are hungry, reducing food loss and waste is critical to creating a world with zero hunger and achieving the Sustainable Development Goals by ensuring sustainable consumption and production patterns. This study analyses how scientific research concerning food loss has evolved in recent years, with a focus on the supply chain of agri-food companies. Bibliometric techniques were used to analyse a sample of 181 publications from journals indexed in the Web of Science from 2012 to 2021. The obtained results show a growing interest in this topic and a clear concern for the management and prevention of food loss. An important conclusion is that a holistic approach from a supply chain perspective is needed to devise food loss reduction strategies focusing future lines of research on stakeholder collaboration, the circular economy and related regulatory changes. The study has implications for entrepreneurs and decisionmakers due to the effect that a reduction in food loss has on business strategies, as well as for policymakers in order to create updated food safety and quality regulations.

## 1. Introduction

Food loss and waste (FLW) has become one of the great paradoxes of our global society, with numerous environmental, economic and social implications [1]. On the one hand, food security is compromised as millions of people worldwide go hungry and, on the contrary, millions of tons of food are wasted [2]. According to the Food and Agriculture Organization of The United Nations (FAO), it is estimated that one-third of food is lost or wasted [3,4]. There are different definitions of food loss and food waste, which has sometimes been a problem for comparing studies and even for unifying results [5]. The FAO is the body that defines food loss and food waste with a focus on the food security dimension [6]. Food loss refers to the reduction in the mass of edible food along the supply chain for human consumption [7]. Some researchers think that food loss and waste occurs throughout the food supply chain [8,9], while some researchers propose that food loss occurs in the upstream supply chain and food waste happens in the downstream supply chain [10]. However, in our research we will go with Antonelli et al. [10]. On the other hand, food waste occurs when food produced for consumption is discarded or not consumed by humans (food is spoiled or was edible when discarded) [11]. Food loss occurs in the upstream supply chain (production, post-harvest or processing) [12], while food wastage occurs downstream, at the retail or consumer stages, where product aesthetics and quality standards to be met play a major role [13]. 

In addition, many natural resources are consumed in food production, so food loss has an environmental impact in terms of freshwater use, soil nutrient depletion and greenhouse gas (GHG) emissions [11,12,13,14,15,16,17]. This misuse of resources makes it a crucial global problem that is present in the United Nations Sustainable Development agenda. Specifically, Sustainable Development Goal (SDG) 12.3 aims to halve food loss and waste by 2030 [18]. Food loss and waste is a global problem with major consequences at all levels [19].

This challenge has been echoed by the scientific community, and there has been a large amount of research on food waste on the demand side [20]. Investigations from the demand side point of view take into account consumer behaviour in relation to the food they consume, the preservation of food and the waste generated [18,19,20,21,22,23,24,25,26,27,28,29]. On the supply side, there are also numerous studies, although they have focused primarily on quantifying food loss [21,22,23,24,25,26] and analysing the supply chain as a whole (wholesalers, retailers or distributors) [24,25,26,27,28,29]. Therefore, there is scope for analysis at other levels and, above all, for the identification of loss hotspots, possible causes and solutions to be implemented [30].

**Figure 1:**
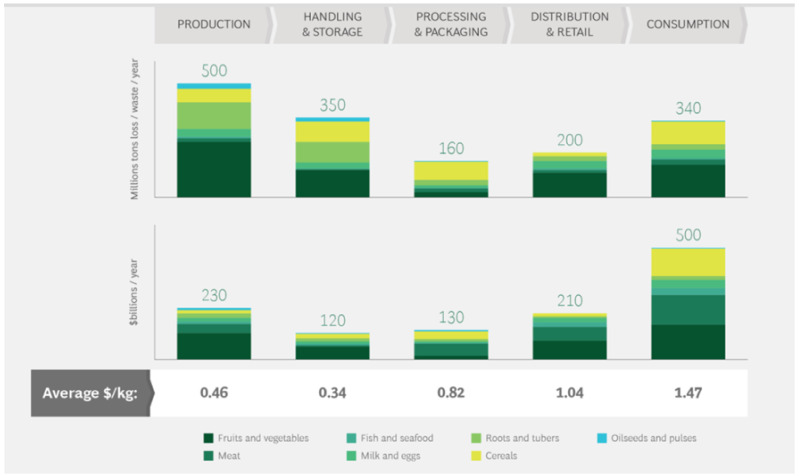
Food loss and waste occur across the value chain. Source: [31].

Food loss or waste occurs at all stages of the supply chain, but there are more losses during production and consumption [28,29,31,32] (Figure 1). In developing countries, the hotspots of loss are at the beginning of the supply chain, in the production and transport of food from farms, mainly due to a lack of both technical and financial resources [14], while in developed countries, waste in the later stages of the supply chain (consumption) is often more important, taking into account both retailers and consumers [33].

The agri-food system is composed of all farm-level procedures and the relationships of all actors involved at each level of the supply chain [7]. The term agrosystem was defined by Conway [34], in his explanation of the agricultural production process and how it is composed of various stakeholders. In 2017, Horton [35] extended this term and showed that food loss occurs at all points in the system. The causes can be varied [24] and can be related to harsher climates [36], nutrient-deficient soils [37], non-optimal storage [38], inadequate transport [39], etc.

None of these models have delved into the causes of food loss that would really help to reduce them and achieve improved efficiency and sustainability in the supply chain [7].

Clearly, agri-food companies play a key role in the food supply chain and can be catalysts in reducing food loss [40]. In addition, businesses should be aware that addressing this social, economic and environmental challenge will be rewarded through tangible business benefits (lower costs, new revenue opportunities and improved reputation) [31,41]. Many companies already make use of such initiatives that enhance their brand and increase their ability to attract and retain talent [31].

The aim of this study was to understand the evolution and relationship between the terms food loss and agri-food industry supply chain. To this end, a bibliometric analysis was developed that aims to address gaps in the literature (the need to focus future research on supply chain management to minimise food loss and to know how to manage the loss that is unavoidable), as well as to indicate possible trends that drive the reduction in food loss in primary production and are not sufficiently developed. Our research has implications for entrepreneurs and managers as it highlights the effect that supporting food loss reduction can have on business strategies, as well as the economic, social and environmental benefits. It also serves to guide future scientific research in this field.

The document presented has the following structure: Section 1, where the justification for this research is presented; Section 2, where the bibliometric techniques used are described, as well as the materials and software; Section 3, in this section, the most important issues of the bibliometric analysis related to food losses in the value chain of agri-food companies are described. This section analyses the key aspects provided by the researchers through a systematic review of the literature, ending with the Section 4 and Section 5.

## 2. Materials and Methods

### Data Collection and Methology

**Figure 2:**
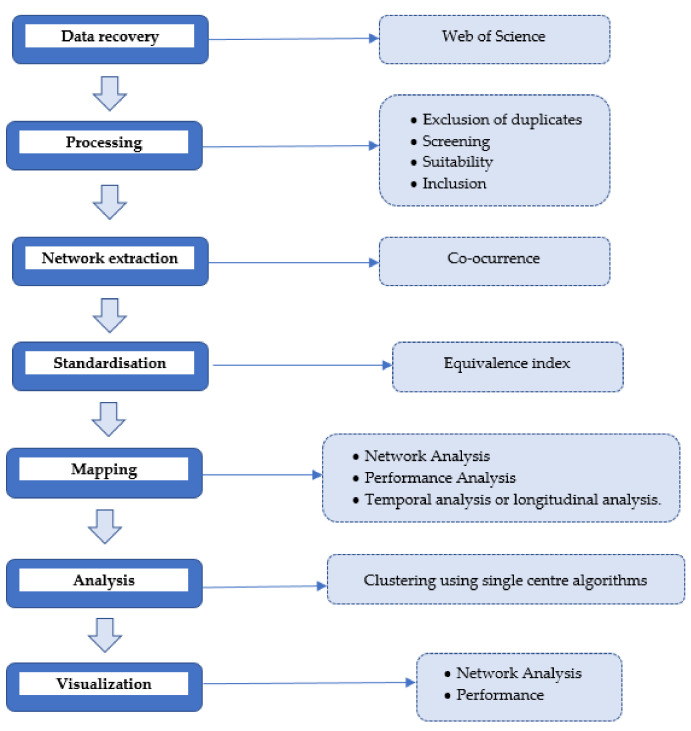
Schematic of general workflow in scientific mapping. Adapted from [42].

According to Cobo et al. [42] the scientific mapping analysis of a research area is performed in consecutive steps: data retrieval, preprocessing, network extraction, normalisation, mapping, analysis and visualisation (Figure 2).

1st Step—Data Retrieval: We collected published papers on the topic from the Web of Science (WOS) database. The Web of Science database was used for the bibliometric analysis, specifically, the main collection of the database. This database is a collection of more than 68 million documents from 1900 to the present day. We used an advanced search, entering the field tags “food loss”, “supply chain” and “agri-food companies” in the title (TI), in the author keywords (AKs), in the keywords plus (KP) and in the abstract (AB). The search was conducted on 15 March 2021. It included the Science Citation Index—Expanded (SCI-E), the Social Science Citation Index (SSCI) and the Emerging Sources Citation Index (ESCI). The period chosen for the study was from 2012 to May 2021, obtaining 195 references.

2nd Step—Preprocessing: The units of analysis used were the keywords (authors’ keywords, journal keywords, indexing keywords such as any combination of keywords) presented in the selected documents.

**Figure 3:**
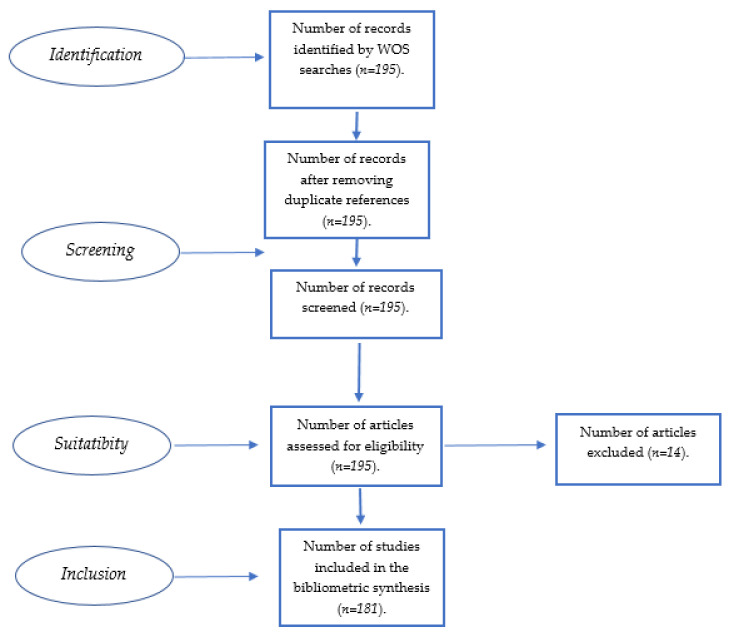
Prima flow diagram [43].

Using the PRISMA flow diagram [43], from the 195 citations obtained, no references were excluded in the screening as the search was very focused and was carried out in a single database. Finally, by the suitability process, 14 references were excluded as food loss in agri-food companies was not the main focus of the paper (Figure 3).

At the end of this 2nd step, we had a total of 181 references for the bibliometric analysis.

3rd Step—Network Extraction: The relevant information was extracted from the data, which included the co-occurrence frequencies of the keywords.

4th Step—Normalisation: By selecting the similarity measure, in our case, the equivalence index, we normalised the network. The similarities between the elements were calculated according to the frequencies of the co-occurrence of keywords.

5th Step—Mapping: Through a clustering process, we approximated subgroups of keywords that were strongly linked, implying that they corresponded to centres of interest or research problems studied by researchers [44]. Simple centre algorithms were selected that automatically returned labelled clusters, so there was no need for further processing to label the clusters [42].

**Figure 4:**
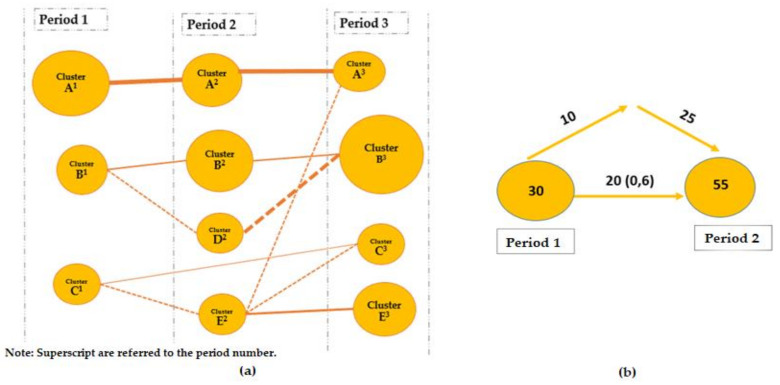
Thematic evolution map (**a**) and overlap map (**b**). Source: adapted from [42].

**Figure 5:**
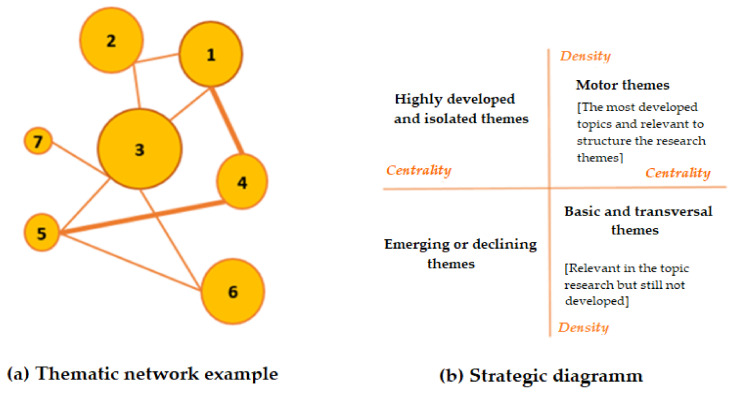
Thematic network (**a**) and strategic diagram (**b**). Source: adapted from [42].

6th Step—Analysis: The following analyses were performed on the generated maps:

(A) The map of the evolution of the themes (Figure 4a) was made up of as many columns as periods created in the study, and in this map, we can see the topics most covered by science in each year, linked together according to the evolution of these topics. Figure 4b shows an example of the overlapping map showing the maintenance or entry and exit of keywords over the periods.

(B) To understand the situation of the most discussed topics in a given period, the strategy map (Figure 5b) was divided into four zones according to centrality and density. Centrality measures the relevance of the external connections of the theme under study with other themes. Density refers to the level of internal cohesion of the group under investigation, i.e., it is the internal pressure of the keywords of the theme. In the upper right quadrant (Figure 5b) are the topics that were most discussed and developed in the period. In the lower right corner (Figure 5b) are the topics that have scarcely been developed but have made important contributions to the research analysed. In the lower left-hand corner (Figure 5b) are the topics that are not yet developed and may be emerging as attractive topics for research. Finally, in the top left corner (Figure 5b) are those topics that are very specific and isolated [42].

The thematic network (Figure 5a) represents the graph formed by the keywords and their interconnections within a theme. It is be labelled with the name of the most central keyword within the topic. The volume of the spheres varies according to the number of documents for each keyword, and the thickness of the link between the spheres is proportional to the equivalence index or internal relationship between the two concepts [42].

7th Step—Visualisation: The results of the temporal or longitudinal analysis shown in the evolution map and a graph of overlapping elements, helped to detect the evolution of the groups throughout the different periods and to study the transitory and new elements of each period, as well as the elements shared by two consecutive periods. All of this is discussed in the following section.

## 3. Results

**Figure 6:**
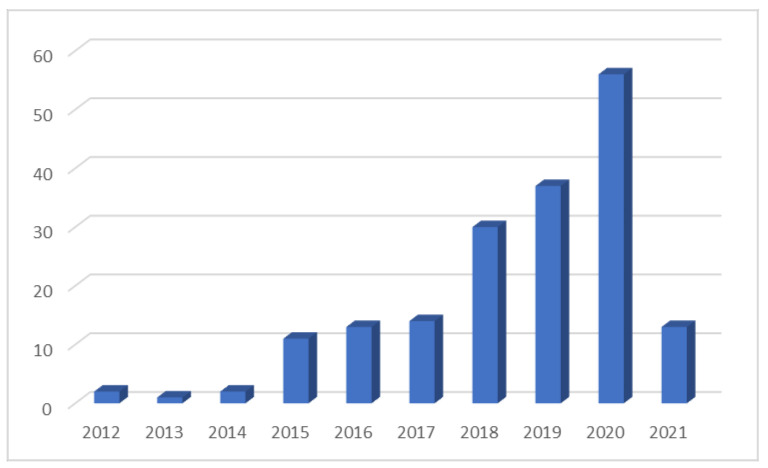
Number of documents per year of publication.

We analysed 181 articles published between 2012 and 2021. From 2017 onwards, the publication of articles followed an upward trend, which demonstrates the interest in the subject among the scientific community (Figure 6).

Table 1 shows the journals with more than four documents included in the present study. The most used journals were Journal of Cleaner Production and Sustainability, with 15 documents in each journal.

Table 2 shows the number of citations of the main articles, as well as the year of publication. The most cited paper was “Total and per capita value of food loss in the United States” [24] published in the journal “Food Policy”.

### 3.1. Evolution of Keywords

**Figure 7:**
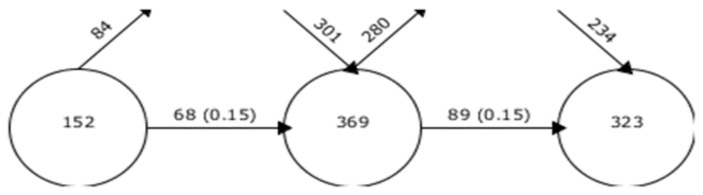
Overlapping graph of keywords from 2012 to 2021.

To analyse the development of the current research field in relation to food loss in the supply chain of agri-food companies, it is useful to represent how the keywords used in the research papers have evolved over time in the different periods. Thus, in the overlapping map (Figure 7), the circles represent each period, and the number of each circle represents the associated keywords in that period. The outgoing top arrow represents the keywords that have disappeared from one period to the next, and the incoming top arrows indicate the keywords added to the new period. The arrows connecting the periods give the number of keywords shared between them, including the stability index between them.

The first period (2012–2017), although it covers a period of 6 years, was characterised by a smaller number of keywords than the last two periods (2018–2019 and 2020–2021), both of which actually had somewhat similar numbers. In the first period, there were 152 keywords, of which 84 were no longer used in the following periods. Of the words used in this first period, 68 were also carried over to the second period. For the second period, 301 additional words were introduced, totalling 369 keywords, an increase of 142%. From the second to the third period, 280 keywords disappeared, 234 new ones were introduced and 89 moved to the last period. The number of keywords in the third period was significantly higher than in the first period, although there was a decrease in the number of words from the second to the third period. The stability index between the three periods was the same (0.15), so the subject matter was undergoing a significant evolution, and there was still no similarity between periods as new subjects and concepts were introduced. Furthermore, from 2018 onwards, the number of works on these subject increased, which would justify the significant increase in the number of keywords. Between the second and third periods, the number of words was more homogeneous.

### 3.2. Thematic Evolution Map

**Figure 8:**
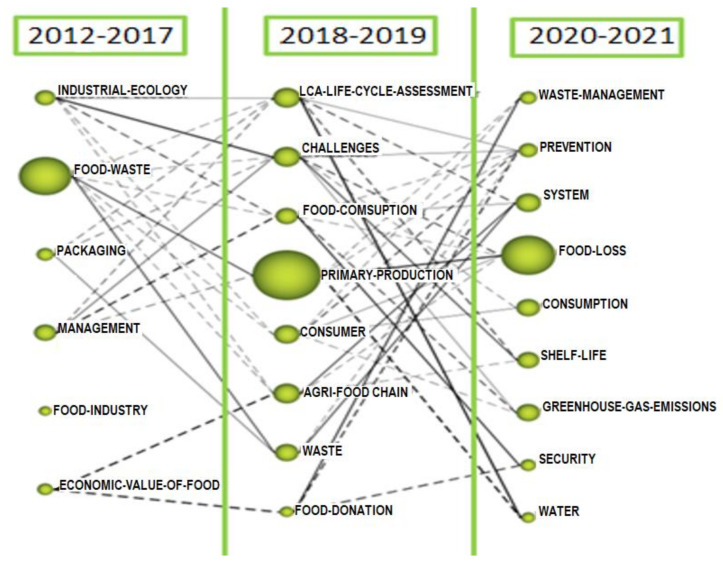
Thematic evolution map.

Within the longitudinal view, the evolution map (Figure 8) shows in columns the different periods of the sample, under which the most relevant themes can be found in clusters. These clusters are connected across the periods by lines, which represent the point evolution of the topics. If two clusters are linked by a continuous line, they share a main theme, but if two clusters are linked by a discontinuous link, it means that they share elements but not a main theme. Some clusters may not be connected by lines, in which case, they are emerging or isolated themes that have no connection with any other cluster at the moment and whose evolution over the different periods should be followed. The size of each cluster depends on the selected performance measures. In the case of our study, we considered the average number of citations.

Regarding the evolution of the topics by periods (Figure 8), it was found that the concept of “food loss” was consolidated over time. In the period 2012–2017, the concept “food waste” had the highest number of citations. This period was dominated by scientific research related to the quantification of food loss and food waste [24,25,26]. In addition, there was concern about the relationship that packaging may have on food loss [47], as well as the management of losses, including the economic valuation of loss [29,32,46,49].

In the 2018–2019 period, the concept of “primary production” [52] appeared strongly and shared the same theme as the concept of “food waste” in the first period. In these years, there was a large amount of interest in studying food waste on farms [53], with studies of different crops: potatoes [54], fresh fruit and vegetables [52,53,55,56] and tomatoes [1], among others. The aim was to provide producers with strategies to increase the amount of fresh produce in the supply chain and to make production more sustainable, reducing the impact of agriculture on the environment [2,50,53]. Hence, other concepts prominent in this period included “agri-food chain” [16,19,42,45], which shared elements with the concept of the economic valorisation of food waste from the first period and placed importance on collaboration between producers and cooperatives to reduce food loss and enhance farm yields. Another prominent concept was “challenges”, which was directly related to previous concepts, such as “management” and “industrial ecology”, and highlighted the challenges facing the agri-food sector [26,29,54,55,57,58], as well as “food donation” as a way of not wasting food and taking social action [59]. There was also interest in the concept of “LCA—life cycle assessment”, which focused on the study of food losses in the context of the life cycle of the supply chain, particularly in reference to the circular economy [54,57,60].

The 2020–2021 period represents the consolidation of the concept of “food loss”, which stemmed from a direct linkage of the earlier-period concepts “food waste” and “primary production”. Of particular importance was the application of different perspectives and methodologies in food loss studies [61], the need for collaboration between stakeholders in the supply chain [62] and the need for a holistic supply chain approach [63] that takes into account the different food categories and stages of the supply chain to facilitate the decision-making process [64].

In addition, the concept “system” [27,30,62,65] emphasised the consideration of waste as an intrinsic element of food systems. Other concepts to be highlighted were “waste management” and “prevention”, which were directly and indirectly related, respectively, to the food donations that appeared in the second period, and which emphasised the need for the prevention and use of production surpluses or donations to alleviate food insecurity [66].

The concepts of “shelf life” [64,65,67,68] or “greenhouse gas emissions” [66,67,69,70] were part of the challenges faced by companies and public institutions in the face of the need to reduce food loss already announced in the second period.

### 3.3. Strategic Map and Thematic Network

Longitudinal analysis has made it possible to determine the evolution of the concepts between periods. In the following subsections, we analyse the importance of each subject in the research field for each of the periods.

#### 3.3.1. Period 2012–2017

**Figure 9:**
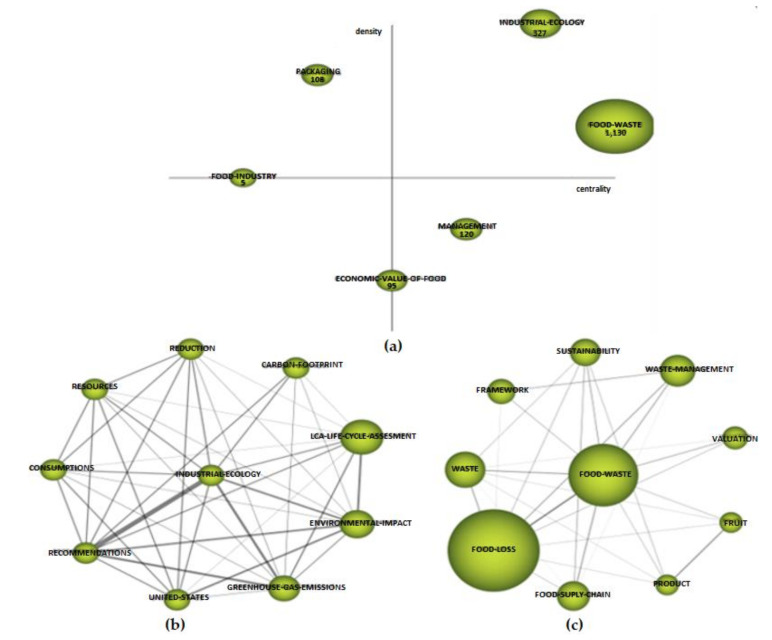
Strategic diagram: (**a**) main thematic network; (**b**,**c**) 2012–2017 period.

Table 3 presents the properties of the clusters, taking into account the centrality and density scores. In turn, in Figure 9a, which represents the strategy diagram for this first period, the number appearing in each cluster sphere represents the sum of citations that each cluster had. The main themes of this period are shown in Figure 9b,c.

In the strategic diagram (Figure 9a), we can see the driving themes (upper right quadrant). In this period, these driving themes were “industrial ecology” (Figure 9b) with 327 citations, a centrality of 89 and a density of 99.19 (Table 3) and “food waste” (Figure 9c) with 1130 citations, a centrality of 123.68 and a density of 32.3 (Table 3). These were the most prominent themes that drove research in this period. The basic theme in this period was “management” located in the lower right quadrant (Figure 9a), which was a transversal theme throughout the scientific production of this period. In the upper left quadrant, we find the cluster “packaging”, which was a more developed or isolated theme with marginal importance for the field of study as it had practically no important external links. On the border between the upper left quadrant and the lower left quadrant (representing emerging or declining themes), we find the cluster “food industry”, which will evolve into an isolated theme as in the thematic evolution map it had no relationship with the rest of the clusters. Likewise, the cluster “economic value of food” was on the borderline between basic and cross-cutting themes and emerging or declining themes. In the thematic evolution map, this cluster was related to two other clusters and will, therefore, evolve indirectly through them.

With regard to the analysis of the thematic network of the two driving themes, in the case of “food waste” (Figure 9c), the keywords with which it was related can be seen, highlighting its relationship with “food loss”, which had an internal link of 0.36 and was the concept with the highest number of citations; therefore, it is the most important in this thematic network. In terms of the number of citations, “waste”, “waste management”, “sustainability” and “food supply chain” also stood out. In relation to the second driving theme, “industrial ecology” (Figure 9b) had a very important relationship—an internal link of 1—with the concept “recommendations”. In terms of the number of citations, “LCA—life cycle assessment”, “environmental impact” and “greenhouse gas emissions” stood out. The latter two had an important internal link with “recommendations”.

#### 3.3.2. Period 2018–2019

**Figure 10:**
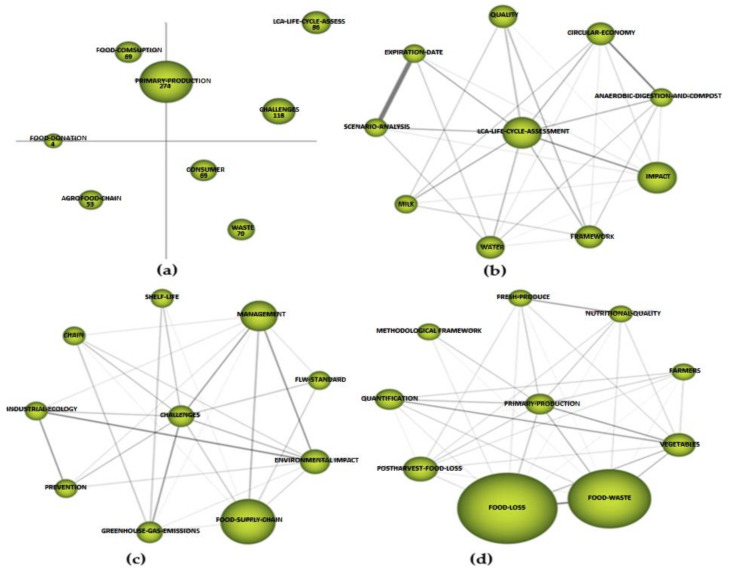
Strategic diagram: (**a**), main thematic network; (**b**–**d**) 2018–2019 period.

In the strategic diagram (Figure 10a), we can see the driving themes of this period (upper right quadrant). In this period, these driving themes were “challenges” with 118 citations and a centrality of 137.62 and a density of 33.67 (Table 4) and “LCA—life cycle assessment” with 86 citations and a centrality of 163.53 and a density of 43.5 (Table 4), being the most prominent themes that drove research in this period. The situation of the “primary production” cluster, halfway between the driving themes and the more developed and isolated themes, is noteworthy. It stood out for its number of citations (274), with a centrality of 113.78 and a density of 35.23 (Table 4). We have already seen in the graph of thematic evolution (Figure 8) that it was the cluster with the greatest weight in this period in terms of number of citations and had a direct relationship with the “food waste” cluster in the first period and with the “food loss” cluster in the third period, which were, respectively, the most prominent in each of the study periods.

The basic themes in this period were “consumer” with 69 citations and “waste” with 70, located in the lower right quadrant (Figure 10a) and were cross-cutting themes throughout the scientific production of this period. In the upper left quadrant, we find the cluster “food consumption”, which was a more developed or isolated theme with marginal importance for the field studied as it had practically no important external links. On the border between the upper left quadrant and the lower left quadrant (representing emerging or declining themes), we find the cluster “food donation”, which, following the thematic evolution map (Figure 10), will develop through clusters such as “waste management”, “prevention”, or “security”. Likewise, the cluster “agri-food chain” was among the emerging or declining thematic clusters. In the thematic evolution map (Figure 10), this cluster was directly related to the “system” cluster, which was a driving theme in the third period.

Regarding the analysis of the thematic network of the two driving themes, in the case of “challenge” (Figure 10c), we can see that the relationship it maintained with the rest of the keywords in the network was quite homogeneous—0.12 in most cases. These concepts represented the different challenges of the topic of study: “prevention”, “environmental impact”, “management” and “greenhouse gas emissions”, among others. In terms of the number of citations, the concepts “food supply chain”, “management” and “environmental impact” stood out. In relation to the second driving theme, “LCA—life cycle assessment” (Figure 10b) was related to “impact” through an internal link of 0.3. Additionally, the relationship between the concept “circular economy” and “impact” was noteworthy due to the number of citations. There was an important internal relationship between the concepts “expiration date” and “scenario analysis”. We find the analysis of the cluster “primary production” (Figure 10d) interesting due to its weight in terms of number of citations as well as its centrality and density. In this cluster, we find an almost identical internal relationship between “primary production” and “food loss” and “food waste”, respectively. The relationship between these clusters is evident both in the thematic network analysis and in the thematic evolution map. Additionally, the internal link between “food loss” and “food waste”, which was 0.46, as well as the number of citations of each of these concepts, is noteworthy.

#### 3.3.3. Period 2020–2021

**Figure 11:**
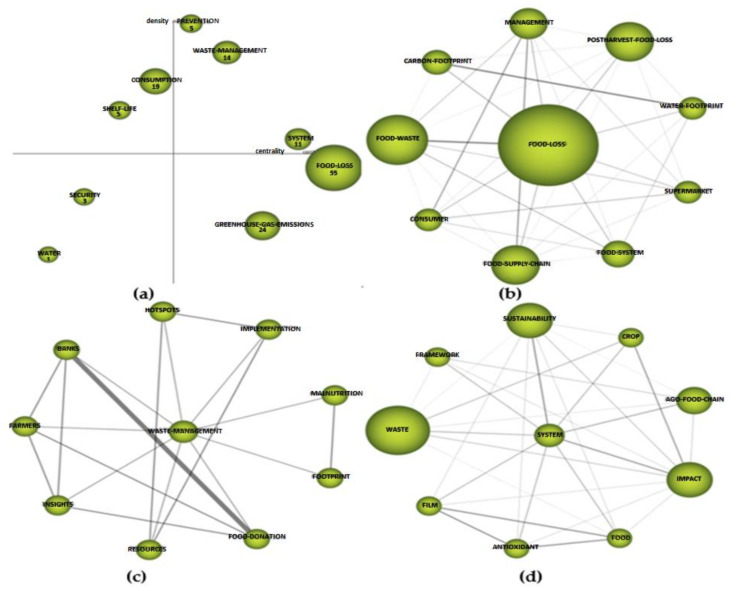
Strategic diagram: (**a**) and main thematic network; (**b**–**d**) 2016–2021 period.

In this period, the driving themes were (Figure 11a) “waste management” with 14 citations and a centrality of 129.04 and a density of 47.5 (Table 5), “system” with 11 citations and a centrality of 138.03 and a density of 68.03 (Table 5) and “prevention” with 5 citations and a centrality and density of 116.92 and 68.03, respectively, which were the most prominent and driving themes of the research in this period. The difference in citations compared to the driving themes of the first period is evident. This may be due to the fact that the latter period is the most recent, and many of the studies referred to were published in 2021. The basic themes in this period were “greenhouse gas emissions” located in the lower right quadrant (Figure 11a) and “food loss”, for which it should be noted that the number of citations (55) was between the area of the driving themes and that of the basic themes. Due to the weight of this cluster in the thematic evolution map (Figure 8), its future development will occur towards the motor themes. In the upper left quadrant, we find the clusters “consumption” and “shelf life”, which were the most developed or isolated themes with a marginal importance for the studied field, as they had practically no important external links. In the lower left quadrant (representing emerging or declining themes), we find the “security” and “water” clusters.

Regarding the analysis of the thematic network of the driving themes, in the case of “waste management” (Figure 11c), the keywords to which it was related were “farmers”, “resources”, “implementation”, “banks” and “food donations”, among others. It had the same level of internal relationship (0.17) with all these keywords, which all had practically the same weight in terms of the number of citations. It is worth highlighting the relationship between the concepts “banks” and “food donations” with an internal relationship of one, as they are closely related concepts. In relation to the second driving theme “system” (Figure 11d), the related keywords were “waste”, “sustainability”, “impact” and “agri food chain”, among others. These four words had the highest number of citations, although all the words in the network had a similar internal relationship with “system”. Due to the position occupied by the cluster “food loss” due to its centrality and density, we consider its thematic network to be noteworthy (Figure 11b). The words in the network with the highest number of citations were “food waste”, “post-harvest food”, “food supply chain” and “management”. We highlight the internal relationship with “food waste”, which was 0.34. Between “carbon footprint” and “water footprint”, there was also an important internal relationship of 0.33.

## 4. Discussion

As mentioned above, the aim of this research was to understand the evolution and relationship between the terms food waste and the supply chain of agricultural companies. To this end, a bibliometric analysis was carried out in order to respond to the gaps found in the literature, as well as to indicate possible trends that drive the reduction in food loss in raw material production that are not sufficiently developed.

The analysis shows that the scientific debate in this area has transitioned from a small number of contributions in 2012 to becoming a topic of debate with an increasing number of research studies from 2017 onwards, with the year 2020 having the greatest impact. This has been helped by the growing importance of the significant contradiction at all levels and in all areas—food waste and food insecurity. It is estimated that one-third of all food produced worldwide is lost or wasted in a world where almost one billion people go hungry [71]. Reducing food loss and waste is key to creating a world with zero hunger and achieving the Sustainable Development Goals, especially SDG 2 (Zero hunger) and SDG 12 (Ensure sustainable consumption and production patterns).

We have seen that, in the first years of scientific development, there was a more quantitative vision, where the valuation of food losses was very common [24,25,26]; the driving theme was the cluster “food waste” [46], closely related to “food loss” [48]; and the food supply chain was already present in a continuous search for the quantification of loss at the different levels of the supply chain [32]. In addition, there are many studies from the point of view of “industrial ecology” that attempt to provide solutions from the environmental profile of the supply chain [72], estimating greenhouse gas emissions related to food loss and waste [29,32,42,45,70,73] or modelling food loss in life cycle assessment [48]. Every dollar invested in reducing food loss saves USD 14 in operating costs [57].

From 2018 to the early 2020s, a range of work was carried out within the “LCA—life cycle assessment” cluster: adopting a “circular economy” approach to food loss and waste [57]; research into the best packaging for dairy products that takes into account environmental implications and the circular economy [74]; life cycle assessment to evaluate food recovery strategies [58] across all stages of the life cycle [75]. Much of this research is related to the food industry’s “challenges” in terms of food loss, which form another of the most important clusters of this period: packaging-related strategies to save food [76], highlighting the need to focus on reducing loss in those parts of the supply chain, cultivation and supply, where loss is greatest [57]. The concept of “primary production”, which focuses on the various accepted strategies and approaches and the potential for intervention in relation to food waste [27], advocates a solution to the problem of on-farm food loss as environmental risks increase as food waste moves downstream [37] and the need for the further decentralised management of food loss, taking into account the different stages in the supply chain [77]. This is due to the fact that each food or product has very specific reasons for having losses collected at each stage of the supply chain [78].

In more recent years (2002–2021), researchers have oriented their work towards addressing food loss by focusing on the hotspots of loss within industrial processing, taking into account sustainable operations [30], the challenges related to the reuse of food losses either as a donation or other alternatives [66] and above all taking into account a holistic supply chain perspective approach to devise food loss reduction strategies [63]. Collaboration between the different stakeholders in the supply chain encourages mutually supportive relationships [62] and taking a systemic and realistic perspective to address the problem of food waste, not forgetting the need to examine food waste from a societal perspective that relates it to customs and behaviours rather than considering it only as a material object [29,79].

## 5. Conclusions

In conclusion, the relationship between the terms food loss and supply chain is complete. We started from this research objective and throughout the literature review we have seen the importance of intervening in the prevention of food loss on farms and in the first links of the supply chain in order to reduce the environmental risks of food loss. In addition, for more perishable products, stakeholders in the supply chain need to be closely integrated, with mutual commitment to reduce processing, transport and storage times. This will generate greater benefits for all and significantly reduce the percentage of losses of these products.

It is also very important to generate synergies that focus on extending the shelf life of the products, introducing preservation processes in the production line that reduce the risk of losses, as well as optimising costs. The use of technology and fluid communication between all parties involved will increase productivity in the other stages of the supply chain, reducing food losses. The utilisation of unavoidable losses by establishing donations for disadvantaged groups, for the production of by-products or for use as feed on the farm makes the concept of circular economy interesting in this area.

The supply chain of the agri-food industry is the key area where work should be conducted in order to effectively reduce food losses. Over the years, research has shifted from a focus on loss assessment and quantification, in conjunction with the application of environmental recommendations, to striving for appropriate, individualised management at each stage of the supply chain for each product in order to minimise losses in the agri-food industry. In addition to efficiently managing loss for reuse, this process must be carried out with a holistic vision to analyse the current situation. Said management must involve the collaboration of stakeholders throughout each stage of the supply chain in order for food waste to be considered as an element of food systems and to improve knowledge of the requirements demanded by end consumers. Reducing food waste is not only necessary to avoid the associated environmental impacts it causes or to solve the problem of global food insecurity, but it is also beneficial for the economic sustainability, public image and reputation of companies. 

### 5.1. Theoretical Implications

This study has increased the knowledge of food loss by analysing all the articles in the Web of Sciences database up to 15 March 2021, for a total of 181 articles. The results of the analysis provide an order of the topics that have been researched for the study period, from 2012 to 2021, classified by thematic groups or clusters. The maps provided by the SciMAT programme (the longitudinal map, as well as the strategic diagram and the main thematic network maps), help to clearly visualise the evolution of the research topics and the connections between them. This will enable food loss researchers to guide future research with knowledge of the current context of the publications in the Web of Science database.

### 5.2. Practical Implications

Our research has implications for business and decisionmakers as it highlights the impact that supporting food loss reduction can have on business strategies, as well as the economic, social and environmental benefits. It also has implications for policymakers due to the importance of incorporating advances in food loss reduction strategies in food safety and quality regulations. It also serves to guide future scientific research in this field.

### 5.3. Limitations and Future Lines of Research

The main limitation of our work comes from the choice of the Web of Science Core Collection as the reference database. We understand that, for future analyses, the use of several sources of information can complement the results presented here. Furthermore, interpreting the structure of a research field is highly complex. Despite the detailed study of all the research included and the structured analysis followed in this study, there may be some unintentional bias on the part of the researchers.

Future lines of research should be directed towards the further study of the influence of climate change as an inhibitor of food loss reduction, investigating the role of collaboration between stakeholders in the supply chain in cases such as cooperatives, studying whether the vertical integration of agri-food companies influences food loss, and analysing how regulatory changes in food quality and safety may affect the ability of actors to reduce food loss.

## Figures and Tables

**Table 1:** Number of documents ordered by source.

Journal	Documents
Journal of Cleaner Production	15
Sustainability	15
Waste Management	9
Resources Conservation and Recycling	7
Science of the Total Environment	6
Food Policy	4
Journal of Industrial Ecology	4
Horticulture	4
Journal of Agriculture Food Systems and Community Development	4
International Journal of Life Cycle Assessment	4
Food	4

Note: Source: own elaboration based on SciMAT data.

**Table 2:** Most cited documents.

Title	Authors	Year	Citations
Total and per capita value of food loss in the United States [24]	Buzby and Hyman	2012	266
Greenhouse gas emission estimates of U.S. dietary choices and food loss [45]	Heller and Keoleian	2015	135
Food loss rates at the food retail, influencing factors and reasons as a basis for waste prevention measures [46]	Lebersorger and Schneider	2014	99
Packaging’s role in minimising food loss and waste across the supply chain [47]	Verghese, Lewis, Lockrey and Williams	2015	77
The value of food waste: an exploratory study on retailing [25]	Cicatiello, Franco, Pancino and Blasi	2016	76
Modelling of food loss within life cycle assessment: from current practice towards a systematisation [48]	Corrado, Ardente, Sala and Saouter	2017	56
Food waste accounting along global and European food supply chains: state of the art and outlook [27]	Corrado and Sala	2018	47
A half-century of production-phase greenhouse gas emissions from food loss and waste in the global food supply chain [28]	Porter, Reay, Higgins and Bomberg	2016	44
Food waste in Japan: Trends, current practices and key challenges [49]	Liu, Hotta, Santo, Hengesbaugh, Watabe, Totoki, Allen and Bengtsson	2016	40
Diet change and food loss reduction: what is their combined impact on global water use and scarcity? [50]	Jalava, Guillaume, Kummu, Porkka, Siebert and Varis	2016	39
The opportunity cost of animal-based diets exceeds all food losses [51]	Shepon, Eshel, Noor and Milo	2018	36

Note: Source: own elaboration based on SciMAT data.

**Table 3:** Cluster information.

Name	Centrality	Centrality Range	Density	Density Range
Industrial ecology	89	0.83	99.19	1
Food waste	123.68	1	32.3	0.67
Packaging	31.66	0.33	81.25	0.83
Management	50.9	0.67	16.75	0.33
Food industry	14.09	0.17	25	0.5
Economic value of food	34.17	0.5	9.52	0.17

**Table 4:** Cluster information.

Name	Centrality	Centrality Range	Density	Density Range
LCA—life cycle assessment	163.53	1	43.5	1
Challenges	137.62	0.88	33.67	0.62
Food consumption	104.51	0.38	39.19	0.88
Primary production	113.78	0.5	35.23	0.75
Consumer	120.11	0.62	25.02	0.38
Agri-food chain	98.87	0.25	20.98	0.25
Waste	129.22	0.75	12.99	0.12
Food donation	38.14	0.12	29.17	0.5

**Table 5:** Cluster information.

Name	Centrality	Centrality Range	Density	Density Range
Waste management	129.04	0.67	47.5	0.89
Prevention	116.92	0.56	68.03	1
System	138.03	0.89	33.14	0.56
Food loss	192.4	1	28.27	0.44
Consumption	113.76	0.44	35.15	0.78
Shelf life	104.43	0.33	34.17	0.67
Greenhouse gas emissions	137.54	0.78	19.99	0.22
Security	69.18	0.22	22.22	0.33
Water	35.8	0.11	4.44	0.11

## Data Availability

Not applicable.
